# Ixazomib, lenalidomide, and dexamethasone in patients with newly diagnosed multiple myeloma: long-term follow-up including ixazomib maintenance

**DOI:** 10.1038/s41375-019-0384-1

**Published:** 2019-01-29

**Authors:** Shaji K. Kumar, Jesus G. Berdeja, Ruben Niesvizky, Sagar Lonial, Jacob P. Laubach, Mehdi Hamadani, A. Keith Stewart, Parameswaran Hari, Vivek Roy, Robert Vescio, Jonathan L. Kaufman, Deborah Berg, Eileen Liao, S. Vincent Rajkumar, Paul G. Richardson

**Affiliations:** 1 Mayo Clinic, Rochester, MN USA; 2 Sarah Cannon Research Institute, Nashville, TN USA; 3 Myeloma Center, Weill Cornell Medicine, New York Presbyterian Hospital, New York, NY USA; 4 Department of Hematology and Medical Oncology, Winship Cancer Institute of Emory University, Atlanta, GA USA; 5 Dana-Farber Cancer Institute, Boston, MA USA; 6 West Virginia University, Mary Babb Randolph Cancer Center, Morgantown, WV USA; 7 Mayo Clinic College of Medicine, Scottsdale, AZ USA; 8 Division of Hematology Oncology, Medical College of Wisconsin, Milwaukee, WI USA; 9 Mayo Clinic, Jacksonville, FL USA; 10 Cedars-Sinai Outpatient Cancer Center at the Samuel Oschin Comprehensive Cancer Institute, Los Angeles, CA USA; 11 Millennium Pharmaceuticals Inc. (a wholly owned subsidiary of Takeda Pharmaceutical Company Limited), Cambridge, MA USA; 12 Present Address: Biogen Inc., Cambridge, MA USA

**Keywords:** Myeloma, Drug development

## Abstract

Triplet combinations containing a proteasome inhibitor are a standard of care in newly diagnosed multiple myeloma (NDMM). We examined the long-term efficacy and safety of the all-oral combination of weekly ixazomib plus lenalidomide-dexamethasone (IRd), followed by single-agent ixazomib maintenance in NDMM patients. Of 65 enrolled patients, 53 received ixazomib 4 mg (days 1, 8, and 15) plus lenalidomide 25 mg (days 1–21) and dexamethasone 40 mg (days 1, 8, 15, and 22) for up to twelve 28-day induction cycles. Twenty-three patients discontinued induction for stem cell transplantation (SCT). In the remaining 42 patients, overall response rate was 80%, including 63% ≥very good partial response (VGPR) and 32% complete responses. At a median follow-up of 56 months, median progression-free survival (PFS) was 35.4 months in the total population. Twenty-five patients received ixazomib maintenance; eight deepened their response (76% ≥VGPR), and median PFS was 37.2 months in this subgroup. Nine of 42 patients who did not proceed to SCT (14% of total population) had an adverse event requiring discontinuation. Ixazomib (median ≥ 96%) and lenalidomide (median 88–94%) relative dose intensities were maintained throughout treatment. Weekly IRd, followed by ixazomib maintenance, was highly active with acceptable toxicity, enabling long-term administration with no evidence of cumulative toxicities.

## Introduction

Treatment strategies for multiple myeloma (MM) have evolved considerably over the past 15 years, influenced by a variety of different factors [1, 2]. For newly diagnosed MM (NDMM) the use of multidrug combinations for induction therapy has become a standard approach, with phase 3 trials demonstrating improved survival outcomes using triplet regimens compared with doublet regimens [3–5]. This approach has demonstrated benefit both as induction prior to autologous stem cell transplantation (SCT) [3, 6, 7] and in transplant-ineligible patients [5, 8–10]. Another important concept in the field of myeloma has been the increasing use of continuous therapy until disease progression, referred to variably as maintenance therapy or continued initial therapy [11–17]. The SWOG S0777 trial demonstrated prolonged overall survival (OS) when the proteasome inhibitor bortezomib was combined with lenalidomide and dexamethasone (VRd) in transplant-eligible and transplant-ineligible patients with NDMM [5]. In addition to the results of this trial, a recent meta-analysis has demonstrated the beneficial effect of proteasome inhibitors as part of induction therapy [7], leading to VRd being one of the current standards of care for the initial therapy of NDMM [5]. Bortezomib has shown some benefit as maintenance and/or consolidation therapy in some phase 3 trials [18–20]; however, the parenteral administration and risk of peripheral neuropathy (PN) limits its long-term use [15]. Therefore, the need for a convenient and well-tolerated proteasome inhibitor-based frontline therapy that can be dosed for an extended duration of time while providing sustained, deep responses with minimal late-onset or cumulative toxicity remains an unmet need. This is particularly important for subgroups with high unmet need, including the elderly.

The oral proteasome inhibitor ixazomib is approved in more than 60 countries, in combination with lenalidomide and dexamethasone (Rd), for patients with MM who have received at least one prior therapy [21]. The oral administration combined with the tolerability profile of ixazomib reported in patients with relapsed/refractory MM [22–30] demonstrates the opportunity for a proteasome inhibitor-based induction therapy and maintenance therapy that is convenient to administer, yet has a favorable toxicity profile, with limited neurologic toxicity and no long-term or late-onset toxicities. Indeed, recent results from the TOURMALINE-MM3 study demonstrate that 2-year maintenance with single-agent ixazomib, post SCT, significantly prolongs progression-free survival (PFS) with a low discontinuation rate [31].

We previously reported the results of a phase 1/2 study assessing the all-oral triplet regimen of weekly ixazomib plus Rd (IRd) as induction therapy in patients with NDMM [24]. Patients received up to 12 cycles of IRd induction therapy followed by long-term single-agent ixazomib maintenance. Patients considered eligible for autologous SCT could withdraw from the study and proceed to SCT after six cycles of IRd induction. The recommended phase 2 dose of ixazomib was determined as 4 mg weekly, and weekly IRd induction was shown to be active and generally well tolerated in NDMM [24]. The limited follow-up at the time of the initial report (median of 14.3 months) precluded assessment of the benefits and toxicity associated with long-term ixazomib therapy. Here we report the long-term efficacy and safety of IRd induction therapy followed by single-agent ixazomib maintenance in patients with NDMM who did not proceed to SCT.

## Subjects and methods

### Study design

This open-label, dose-escalation, phase 1/2 study evaluated the safety, tolerability, and efficacy of weekly oral ixazomib combined with Rd, followed by single-agent ixazomib maintenance, in patients with NDMM. Patients were enrolled at 10 sites in the United States between November 22, 2010 and February 28, 2012. The study was performed in accordance with the International Conference on Harmonization Guidelines for Good Clinical Practice and appropriate regulatory requirements, and with approval of Institutional Review Boards at individual enrolling institutions. The study was registered at www.clinicaltrials.gov as NCT01217957.

Patients aged 18 years or older with NDMM were enrolled. Patients required measurable disease, Eastern Cooperative Oncology Group performance status of 0–2, and adequate hematologic, hepatic, and renal function. Detailed eligibility criteria are provided in Supplementary Table S1. Patients with: grade ≥ 2 PN; major surgery or infection requiring antibiotics within 14 days; uncontrolled cardiovascular conditions, including uncontrolled hypertension, uncontrolled cardiac arrhythmias, symptomatic congestive heart failure, unstable angina, or myocardial infarction within the past 6 months; prior deep vein thrombosis; prolonged QT interval; and known human immunodeficiency virus or hepatitis infections were excluded. All patients provided written, informed consent prior to participation in the trial.

### Treatment

During induction, ixazomib was administered orally on days 1, 8, and 15 of a 28-day cycle along with standard doses of lenalidomide (25 mg daily on days 1–21) and dexamethasone (40 mg weekly) for up to 12 cycles. Dose modifications of all three drugs were permitted for toxicities suspected to be related to the specific drugs. Relative dose intensity (RDI) over the course of treatment was determined, and was defined as:

$${{RDI}} = 100 \times \left( {\frac{{{\mathrm{total}}\,{\mathrm{amount}}\,{\mathrm{of}}\,{\mathrm{dose}}\,{\mathrm{taken}}}}{{{\mathrm{total}}\,{\mathrm{planned}}\,{\mathrm{dose}}\,{\mathrm{over}}\,{\mathrm{treated}}\,{\mathrm{cycles}}}}} \right)$$

Where the total planned dose was calculated by:

$$\begin{array}{*{20}{l}} {{\mathrm{Total}}\,{\mathrm{planned}}\,{\mathrm{dose}}} \hfill & = \hfill & {\left( {{\mathrm{dose}}\,{\mathrm{planned}}\,{\mathrm{at}}\,{\mathrm{enrollment}}} \right.} \hfill \\ {} \hfill & {} \hfill & { \times \, {\mathrm{number}}\,{\mathrm{of}}\,{\mathrm{planned}}\,{\mathrm{doses}}\,{\mathrm{per}}\,{\mathrm{cycle}}} \hfill \\ {} \hfill & {} \hfill & {\left. { \times \, {\mathrm{the}}\,{\mathrm{number}}\,{\mathrm{of}}\,{\mathrm{treated}}\,{\mathrm{cycles}}} \right)} \hfill \end{array}$$

The number of planned doses per cycle was 3 for ixazomib, 21 for lenalidomide, and 4 for dexamethasone. Patients were allowed to interrupt therapy for stem cell collection any time after three cycles of induction and to proceed to SCT after 6 cycles at the discretion of the treating physician. Patients who proceeded to SCT did not receive further ixazomib therapy and are not included in the present analysis. Patients who completed 12 cycles of IRd induction therapy could proceed to maintenance with single-agent ixazomib, given at the last tolerated dose during induction. Patients discontinued the study for progressive disease (PD) or unacceptable toxicities not controlled with dose modifications.

### Objectives

The primary objectives were to determine the combined rate of complete response (CR) and very good partial response (VGPR), and the tolerability and toxicity of IRd in patients with NDMM. The secondary objectives included determination of overall response rate (ORR; at least partial response [≥PR]), rates of individual response categories, measures of response durability (time to response, response duration, time to progression, and PFS), and OS.

### Assessments

Adverse events (AEs) were graded using the National Cancer Institute’s Common Terminology Criteria for AEs, version 4.02. Myeloma disease response was assessed by the investigators in accordance with the International Myeloma Working Group uniform criteria, incorporating additional categories of minimal response and near CR [32–34]. All responses were reviewed by the sponsor for consistency with the response criteria. Patients proceeding to SCT and those stopping treatment for reasons other than PD had disease assessments at the end of treatment and every 16 weeks thereafter. Patients who progressed had survival assessments every 16 weeks. Cytogenetic testing was conducted per institutional standard at local laboratories in this study; high-risk cytogenetic abnormalities were defined as any of del (17/17p), t(4;14), and t(14;16). Minimal residual disease (MRD) assessment was undertaken in all patients undergoing a subsequent bone marrow examination for response analysis (primarily CR confirmation) by multiparametric flow cytometry. The sensitivity of MRD assessment was 10^−4^. Patients enrolled in the phase 2 portion of the study completed quality of life (QoL) assessment at screening, at the start of each treatment cycle, and at the end of therapy, using the European Organisation for the Research and Treatment of Cancer Quality of Life Questionnaire C30 (EORTC-QLQ-C30) instrument.

### Statistical analysis

The study design tested the null hypothesis H_0_: CR + VGPR rate = 0.35, and the alternative hypothesis of H_1_: CR + VGPR rate >0.35. With 80% power to reject H_0_ if the true CR + VGPR rate is ≥0.5 and a one-sided significance level of alpha = 0.1, the required sample size was 48.

## Results

### Patients

A total of 65 patients were enrolled, 15 to the phase 1 portion of the trial and 50 to the phase 2 portion; a total of 53 patients received ixazomib 4 mg, the phase 2 dose. Among these 65 patients, 23 proceeded to SCT and discontinued induction therapy after a median of six cycles. The remaining 42 patients continued on therapy. Among these 42 patients, 17 patients discontinued during the induction phase, due to various reasons including: AEs (*n* = 9; 14% of total population, *N* = 65), patient withdrawal (*n* = 4; 6% of total population, *N* = 65), PD (*n* = 2; 3% of total population, *N* = 65), unsatisfactory response (*n* = 1; 2% of total population, *N* = 65), and other reason (*n* = 1; 2% of total population, *N* = 65). The remaining 25 patients completed 12 cycles of induction with IRd and continued to maintenance with single-agent ixazomib. At the data cutoff for the present analysis (median follow-up 55 months), 5 patients remained on treatment; 20 patients had discontinued during the ixazomib maintenance phase due to PD (*n* = 19) and patient withdrawal (*n* = 1). Baseline characteristics of the entire trial cohort as well as the 42 patients who did not proceed to SCT and the 25 patients who received maintenance are shown in Table 1.

**Table 1:** Baseline demographics and disease characteristics of all patients, those who did not receive SCT, and those who proceeded to maintenance

	Total (*N* = 65)	Patients who did not proceed to SCT (*N* = 42)	Patients who received maintenance (*N* = 25)
Median age, years (range)	66 (34–86)	68 (34–86)	69 (34–77)
Age ≥65 years, *n* (%)	34 (52)	26 (62)	16 (64)
Age ≥75 years, *n* (%)	12 (18)	10 (24)	4 (16)
Male, *n* (%)	36 (55)	23 (55)	14 (56)
Race, *n* (%)			
White	52 (80)	33 (79)	18 (72)
Black or African American	12 (18)	8 (19)	6 (24)
Asian	1 (2)	1 (2)	1 (4)
ISS disease stage at diagnosis, *n* (%)			
I	28 (43)	17 (40)	14 (56)
II	28 (43)	18 (43)	10 (40)
III	9 (14)	7 (17)	1 (4)
MM subtype, *n* (%)			
IgG	44 (68)	27 (64)	15 (60)
IgA	14 (22)	10 (24)	5 (20)
IgD	1 (2)	1 (2)	1 (4)
Light chain	6 (9)	4 (10)	4 (16)
Median creatinine clearance, mL/min (range)	81.4 (27.8–167.2)	77.0 (28.0–167.0)	79.0 (46.0–167.0)
High-risk cytogenetic abnormalities^a^, *n* (%)	5 (8)	3 (7)	2 (8)
del 17	2 (3)	1 (3)	1 (4)
t(4;14)	1 (2)	1 (3)	0
t(14;16)	2 (3)	2 (5)	1 (4)

*ISS* International Staging System, *MM* multiple myeloma, *SCT* stem cell transplantation

^a^High-risk cytogenetic abnormalities included: del 17/17p, t(4;14), and/or t(14;16) detected by fluorescence in situ hybridization or metaphase cytogenetics

### Disease response and survival

**Fig. 1:**
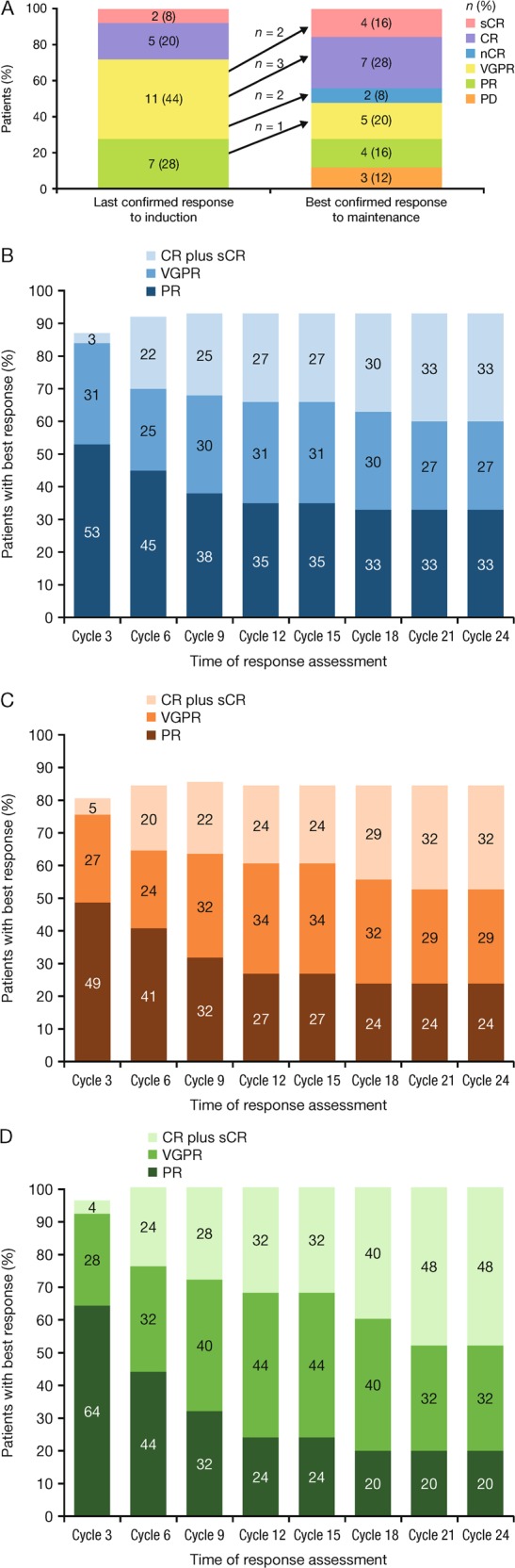
Changes in response rates during induction and maintenance. **a** Deepening of response during the maintenance phase, **b** kinetics of response during induction and maintenance in all patients (*N* = 64), **c** kinetics of response during induction and maintenance in patients who did not proceed to stem cell transplantation (*N* = 41), and **d** kinetics of response during induction and maintenance in patients who received maintenance (*N* = 25)

The best confirmed ORR for all 64 response-evaluable patients (1 patient was not evaluable due to having no post-baseline assessment) was 88%, including 58% of patients with ≥VGPR and 23% with a CR (including stringent CR) (Table 2). Among the 41 response-evaluable patients who did not proceed to SCT, the ORR was 80%, including a 63% ≥VGPR rate and a 32% CR rate. Among the 25 patients who received maintenance therapy, 8 (32%) had a deepening of their response during maintenance (Fig. 1a). The kinetics of response during induction and maintenance are shown in Fig. 1b–d. Ninety-two percent of patients enrolled in the study had cytogenetic results and an evaluable response assessment. In the overall population, 5 patients had high-risk cytogenetic abnormalities; 1 achieved a CR and 3 achieved a PR (1 was not confirmed); these patients were in the subgroup that did not proceed to SCT. Of these 5 patients, 2 continued into the maintenance phase, during which their best response was CR and PR in 1 patient each. Sixteen of 64 (25%) response-evaluable patients were assessed for MRD, of whom 9 had a best confirmed response of ≥CR. Eight patients were found to be negative for MRD. Therefore, in the total study population, 8 of 64 response-evaluable patients (12.5%) were MRD-negative.

**Table 2:** Treatment outcomes and exposure of all response-evaluable patients, those who did not proceed to SCT, and those who received maintenance

	All patients (*N* = 64)	Patients who did not proceed to SCT (*N* = 41)	Patients who received maintenance (*N* = 25)
**Clinical outcome**			
*Best confirmed response*			
ORR (CR + VGPR + PR)	56 (88)	33 (80)	25 (100)
≥VGPR	37 (58)	26 (63)	19 (76)
CR	15 (23)	13 (32)	11 (44)
sCR	6 (9)	4 (10)	4 (16)
PR	41 (64)	20 (49)	14 (56)
VGPR	22 (34)	13 (32)	8 (32)
Near complete response	5 (8)	4 (10)	3 (12)
Minimal response	3 (5)	3 (7)	0
SD	3 (5)	3 (7)	0
PD	0	0	0
Median time to best response ≥VGPR, months^a^	4.9	6.6	8.5
Median time to best response sCR/CR, months^b^	5.6	5.6	5.8
Median PFS, months (95% CI)	35.4 (17.84, 44.12)	29.4 (17.71, 41.13)	37.2 (20.93, 46.00)
Median follow-up for OS, months	56.3	55.2	56.4
Median OS, months	NE	NE	NE
Landmark OS rate, %			
1 year	94	90	100
2 years	89	87	100
4 years	84	82	92
**Treatment exposure**			
Median cycles of ixazomib received, *n* (range)	7 (1–73)	17 (1–73)	41 (15–73)
Cycles of ixazomib received, *n* (%)			
≥8	32 (49)	29 (69)	25 (100)
≥12	26 (40)	25 (60)	25 (100)
≥16	24 (37)	24 (57)	24 (96)
Median relative dose intensity^c^, %			
Ixazomib	96.3	96.3	96.6
Lenalidomide	88.3	90	93.7
Dexamethasone	92.5	83.3	83.3
Patients remaining on treatment, *n* (%)	5 (8)	5 (12)	5 (20)

Patients who proceeded to SCT did not receive further ixazomib therapy and the best response reported did not include response post SCT

*CI* confidence interval, *CR* complete response, *NE* not estimable, *ORR* overall response rate, *OS* overall survival, *PD* progressive disease, *PFS* progression-free survival, *PR* partial response, *sCR* stringent CR, *SCT* stem cell transplantation, *SD* stable disease, *VGPR* very good PR

^a^*n* = 37, 26, and 19 for all patients, those who did not receive SCT, and those who proceeded to maintenance, respectively

^b^*n* = 15, 13, and 11 for all patients, those who did not receive SCT, and those who proceeded to maintenance, respectively

^c^Relative dose intensity = 100 × (total amount of dose taken ÷ total planned dose over treated cycles), where total planned dose was calculated by (dose planned at enrollment × number of planned doses per cycle × the number of treated cycles). Number of planned doses per cycle was 3 for ixazomib, 21 for lenalidomide, and 4 for dexamethasone

**Fig. 2:**
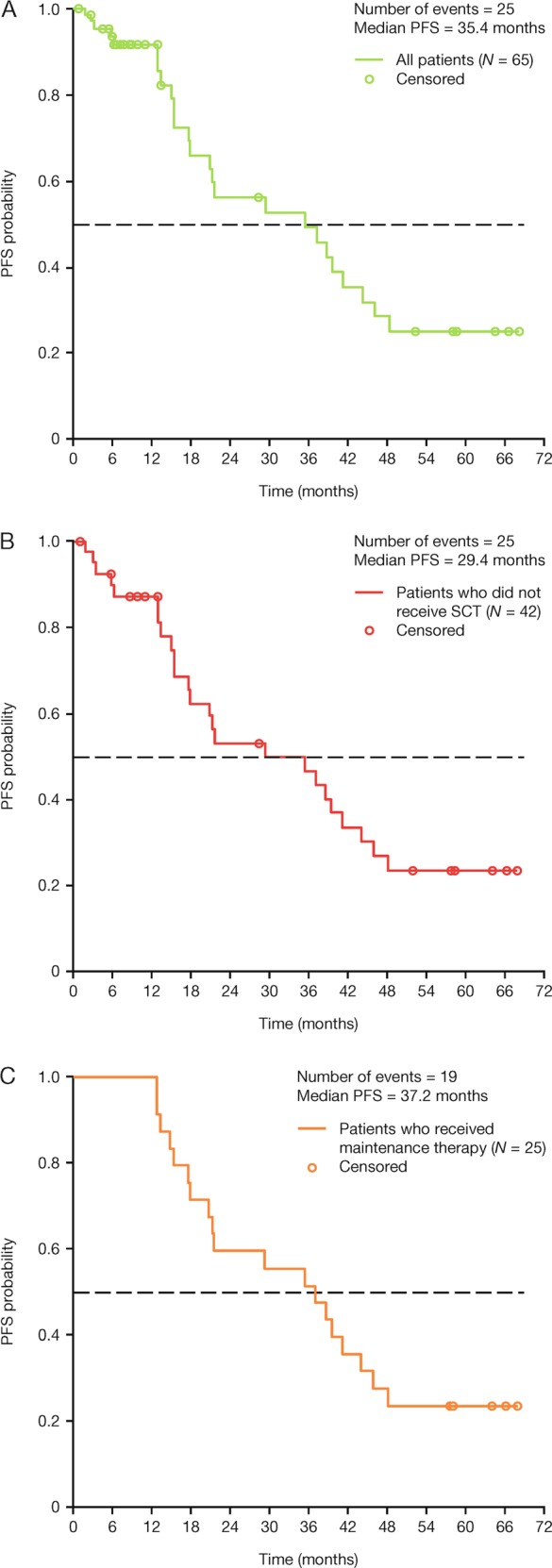
Progression-free survival in **a** all patients*, **b** those who did not proceed to SCT and **c** those who received maintenance therapy. *Patients who received SCT were censored at the time of SCT

With a median follow-up of 55 months, median PFS was 35.4 months (95% confidence interval [CI], 17.84–44.12), 29.4 months (95% CI, 17.71–41.13), and 37.2 months (95% CI, 20.93–46.00) in the total population, in those who did not proceed to SCT, and in those who received maintenance therapy, respectively (Fig. 2). Median OS was not estimable (NE) in any of the three cohorts; however, given the duration of follow-up, estimates of long-term survival are feasible. The 4-year OS rates were 84, 82, and 92% in the total population, those who did not proceed to SCT, and those who received maintenance therapy, respectively (Table 2). Among all 34 elderly patients (≥65 years), the median PFS was 21.4 months (95% CI, 13.37–46.00), and the 4-year PFS rate was 30%. For elderly patients who did not proceed to SCT (*n* = 26) and who received maintenance therapy (*n* = 16), the median PFS was 21.4 months (95% CI, 12.91–46.00) and 37.5 months (95% CI, 15.44–NE), respectively.

### Treatment exposure and safety profile

At data cutoff, with 5 (8%) patients remaining on treatment, patients in the overall population had received a median of 7 cycles (range 1–73) (Table 2). The median RDI was 96.3, 88.3, and 92.5% for ixazomib, lenalidomide, and dexamethasone, respectively. For the 42 patients who did not proceed to SCT, the median number of cycles received was 17 (range 1–73), and median RDI was 96.3, 90.0, and 83.3% for ixazomib, lenalidomide, and dexamethasone, respectively. Among the 25 patients receiving ixazomib maintenance, the median duration of therapy was 41 cycles (range 15–73) and median RDI for ixazomib over the entire treatment period was 96.6%.

The overall safety profile of IRd induction followed by single-agent ixazomib maintenance is summarized in Table 3. Among the 42 patients who did not receive SCT, 86% experienced at least one grade ≥3 treatment-emergent AE, serious AEs were reported in 52%, and 2 patients died on study. Among the 25 patients receiving maintenance, 68% had at least one grade ≥3 treatment-emergent AE during IRd induction, and the incidence was 48% during the single-agent ixazomib maintenance phase. Tolerability in elderly patients (aged ≥65 years) was similar to that in the overall study population; the two on-study deaths that occurred were in patients aged ≥65 years who did not receive maintenance therapy (Table S2). The most common grade ≥ 3 treatment-emergent AEs in the overall population (*n* = 65) included neutropenia (22%), thrombocytopenia (11%), diarrhea (9%), and fatigue (9%) (Table 4). In patients who received maintenance, the overall incidence of grade ≥3 treatment-emergent AEs was higher during the IRd induction phase versus maintenance (68% versus 48%).

**Table 3:** Overall safety profile and most common AEs with IRd induction and single-agent ixazomib maintenance

	All patients (*N* = 65)	Patients who did not proceed to SCT (*N* = 42)	Patients who received maintenance (*N* = 25)^a^	
*n* (%)			AE onset in cycles 1–12 (IRd)	AE onset in cycle 13+ (single-agent ixazomib)
Any grade ≥3 AE	51 (78)	36 (86)	17 (68)	12 (48)
Any serious AE	30 (46)	22 (52)	9 (36)	7 (28)
AE leading to any study drug dose reduction	37 (57)	27 (64)	19 (76)	2 (8)
AE leading to discontinuation of any of the study drugs	10 (15)	9 (21)	0	0
On-study deaths	2 (3)	2 (5)	0	0
Most common AEs (in >25% of the overall study population)				
Diarrhea	39 (60)	28 (67)	14 (56)	13 (52)
Fatigue	39 (60)	27 (64)	18 (72)	2 (8)
Nausea	36 (55)	24 (57)	12 (48)	8 (32)
PN NEC^b^	28 (43)	19 (45)	13 (52)	3 (12)
Upper respiratory tract infection	28 (43)	19 (45)	9 (36)	9 (36)
Constipation	26 (40)	17 (40)	13 (52)	2 (8)
Vomiting	26 (40)	16 (38)	9 (36)	2 (8)
Rashes eruptions and exanthems^b^	23 (35)	15 (36)	11 (44)	3 (12)
Back pain	22 (34)	14 (33)	7 (28)	5 (20)
Peripheral edema	22 (34)	14 (33)	10 (40)	3 (12)
Thrombocytopenia^c^	23 (35)	16 (38)	4 (16)	4 (16)
Insomnia	21 (32)	14 (33)	8 (32)	1 (4)
Cough	21 (32)	17 (40)	8 (32)	3 (12)
Pain in extremity	20 (31)	15 (36)	5 (20)	9 (36)
Dizziness	19 (29)	15 (36)	6 (24)	3 (12)
Neutropenia^c^	20 (31)	12 (29)	5 (20)	0
Pyrexia	18 (28)	13 (31)	6 (24)	2 (8)

*AE* adverse event, *NEC* not elsewhere classified, *PN* peripheral neuropathy, *SCT* stem cell transplantation

^a^Data are split to represent AEs during IRd induction (cycles 1–12), and single-agent ixazomib maintenance; patients could have had a new-onset AE in both treatment periods

^b^Data represent higher-level terms

^c^Pooled terms

**Table 4:** Most common grade ≥ 3 AEs (in >5% of the overall population)

	All patients (*N* = 65)	Patients who did not proceed to SCT (*N* = 42)	Patients who received maintenance (*N* = 25)^a^	
*n* (%)			AE onset in cycles 1–12 (IRd)	AE onset in cycle 13+ (single-agent ixazomib)
Neutropenia^b^	14 (22)	9 (21)	4 (16)	2 (8)
Thrombocytopenia^b^	9 (14)	7 (17)	2 (8)	1 (4)
Diarrhea	6 (9)	4 (10)	0	0
Fatigue	6 (9)	6 (14)	5 (20)	0
Back pain	5 (8)	2 (5)	1 (4)	0
Dehydration	5 (8)	5 (12)	0	1 (4)
Hypokalemia	5 (8)	4 (10)	2 (8)	1 (4)
Lymphopenia	6 (9)	4 (10)	3 (12)	1 (4)
Anemia	4 (6)	4 (10)	2 (8)	0
Hypertension	4 (6)	2 (5)	1 (4)	1 (4)
Hypophosphatemia	4 (6)	4 (10)	3 (12)	0
Leukopenia	5 (8)	5 (12)	1 (4)	0
Rashes, eruptions, and exanthems^c^	4 (6)	4 (10)	3 (12)	0
PN NEC^c^	4 (6)	2 (5)	0	0
Pneumonia	4 (6)	3 (7)	1 (4)	2 (8)
Vomiting	4 (6)	1 (2)	0	0

*AE* adverse event, *NEC* not elsewhere classified, *PN* peripheral neuropathy, *SCT* stem cell transplantation

^a^Data are split to represent AEs during IRd induction (cycles 1–12), and single-agent ixazomib maintenance; patients could have had a new-onset AE in both treatment periods

^b^Pooled terms

^c^Data represent higher-level terms

Among the 42 patients who did not proceed to SCT, treatment-emergent PN of any type was reported in 19 (45%) patients. Most PN events were low-grade, with 17 patients reporting grade 1 or 2 PN; only 2 patients reported grade 3 PN. Among the 25 patients receiving maintenance, there were no cases of new-onset grade 3 or higher PN. There was 1 new primary malignancy, which was not considered related to treatment (squamous cell carcinoma of the skin on the thigh).

Among the patients not proceeding to SCT, AEs led to dose reductions in 27 (64%) patients, of whom 9 (21%), 19 (45%), and 16 (38%) required ixazomib, dexamethasone, and lenalidomide dose reductions, respectively. Overall, the most common treatment-emergent AEs leading to dose reduction were fatigue (19%), PN (12%), diarrhea, insomnia, and weight increase (10% each). AEs leading to discontinuation of study treatment were reported in 9 patients. There were no treatment discontinuations during the maintenance phase (Table 3). Data on QoL were obtained from baseline in patients enrolled to the phase 2 portion of the study. Mean global health status/QoL score from the EORTC-QLQ-C30 instrument over the course of treatment is shown in Supplementary Figure 1.

## Discussion

Proteasome inhibitors are a cornerstone of therapy across the MM treatment paradigm [35], and the combination of a proteasome inhibitor and an immunomodulatory drug remains a standard initial therapy for patients with NDMM [3, 5, 6, 9]. The recent approval of ixazomib in combination with Rd [21] has enabled the development of an all-oral triplet regimen containing both a proteasome inhibitor and immunomodulatory drug, and initial data using this triplet in NDMM patients demonstrated excellent efficacy, safety, and tolerability [24]. The long-term follow-up data presented here not only confirm the efficacy of the all-oral IRd regimen for the initial therapy of MM but also demonstrate the ability to continue therapy among patients not proceeding to SCT, therefore supporting the overall feasibility, efficacy, and tolerability of long-term maintenance with a proteasome inhibitor, namely oral ixazomib.

In the present study, the IRd regimen was associated with an ORR of 88% (58% ≥VGPR, 32% CR) in patients not proceeding to SCT, and a median PFS of 35.4 months in the overall population. These results compare favorably with previous results with the standard-of-care regimen VRd. For example, in the SWOG S0777 trial, which included both transplant-ineligible and transplant-eligible (69%) patients, treatment with VRd resulted in an ORR of 82% (44% ≥VGPR) and a median PFS of 43 months [5]. Similarly, in the EVOLUTION study, which included a majority of transplant-eligible patients (>80%) but also included transplant-ineligible NDMM patients, VRd was associated with an ORR of 85% (51% ≥VGPR) [9]. In the IFM2009 trial, which included transplant-eligible patients only, the ORR with VRd induction followed by lenalidomide maintenance was 97% (77% ≥VGPR) and the median PFS was 36 months [3]. The results with IRd in the present study are particularly notable in the context of these studies when it is considered that over one-third of patients discontinued early to receive SCT and more than half who continued the IRd regimen were aged ≥65 years.

We previously reported on the lack of any adverse impact of this regimen on stem cell collection among patients going to transplant, allowing its use for the initial therapy of transplant-eligible patients [24]. The results reported here show IRd is also an attractive option for patients who are transplant-ineligible or do not desire transplant, as it allows for continued, effective treatment with the same regimen. In the current study, nearly two-thirds of the patients opted not to go to transplant, which is not surprising since over half of the patients were aged ≥65 years, and these patients stayed on therapy for varying durations of time. Among patients not proceeding to SCT, the ORR was 80%, including a ≥VGPR rate of 63%, which is comparable to the overall cohort (88% ORR and 58% ≥VGPR). Among these 41 patients, the median PFS was 29.4 months, and 4-year OS was 82%, again highlighting the efficacy and durability of the IRd regimen when used as initial therapy for transplant-ineligible patients with NDMM. Among the patients aged ≥65 years who did not proceed to transplant, the median PFS was 21.4 months, with a 4-year OS of 28%.

Importantly, in the present study, the IRd regimen was well tolerated in the overall population, in patients who did not proceed to SCT, and in elderly patients, with a similar, generally manageable toxicity profile reported, consistent with prior experience with ixazomib [22–27, 36]. The ability to continue treatment is particularly important for transplant-ineligible patients; here only 9 of the 42 patients who did not proceed to SCT had a treatment-emergent AE requiring discontinuation of any of the study drugs and both the ixazomib and lenalidomide RDIs were maintained during the course of the treatment. One of the advantages associated with ixazomib compared with bortezomib has been the relatively lower incidence of PN, particularly grade ≥3 events. Across the entire trial, PN of any grade was seen in 43% of patients, with very few patients developing grade ≥3 PN (6%). In contrast, VRd has been associated with grade ≥3 PN in nearly 20% of patients in previous trials [3, 9]. Richardson et al. reported sensory neuropathy, motor neuropathy, and neuropathic pain in 80, 18, and 32% of patients, respectively, including at grade 3 in 2, 2, and 3% of patients, respectively [37]. The rate of grade ≥3 neurologic toxicity in the SWOG S0777 trial was 33% [5].

Importantly, the results of this trial highlight the feasibility of single-agent ixazomib as maintenance therapy. Continuous therapy has become a preferred approach, with maintenance post transplant and continued treatment in elderly, non-transplant-eligible patients now routine [14]. Over half of the patients not proceeding to SCT continued on maintenance therapy with single-agent ixazomib. The benefit of single-agent ixazomib maintenance therapy is highlighted by the improvement in the ≥CR rate from 28% at the start of maintenance to 44% as best response on maintenance (Fig. 1a), which translated into prolonged long-term outcomes. Of note, one-third of patients who received maintenance improved their response during the maintenance period. Importantly, when considering the feasibility of continuous therapy, single-agent ixazomib was well tolerated, with few treatment-emergent AEs reported during the maintenance phase and no patients discontinuing therapy during maintenance therapy due to AEs. The overall tolerability profile compares favorably to that observed with continuous Rd therapy in the FIRST trial [11] or lenalidomide maintenance following VRd induction in the SWOG S0777 trial [5]. For example, in the SWOG S0777 trial, 24% of patients required dose reduction of lenalidomide as maintenance, while dose reductions of ixazomib maintenance were required in only 3 of the 25 patients (12%) who proceeded to maintenance, highlighting the ability to maintain the dose intensity of ixazomib maintenance therapy. The median duration of therapy with continuous Rd in the FIRST trial was 18.4 months, whereas in the present study patients who proceeded to maintenance received a median of 41 cycles of therapy (12 cycles of IRd induction and 29 cycles of ixazomib maintenance). Furthermore, in contrast to some reports with lenalidomide maintenance [38], no second primary malignancies considered related to therapy were noted with single-agent ixazomib maintenance in neither the current trial nor TOURMALINE-MM3 [31]. Recently published results of the TOURMALINE-MM3 trial support the use of 2-year fixed duration single-agent ixazomib as maintenance therapy following SCT. The trial showed that ixazomib was an efficacious, well-tolerated, once-weekly oral drug. After a median follow-up of 31 months, there was a 39% improvement in PFS with ixazomib versus placebo (median PFS, 26.5 versus 21.3 months) and a greater rate of deepening of response versus placebo, with little toxicity and maintained QoL. Phase 3 trials of IRd versus placebo-Rd in transplant-ineligible NDMM patients (NCT01850524) and of ixazomib versus placebo maintenance in patients ineligible for SCT (NCT02312258) have completed accrual and the results are awaited.

In conclusion, the IRd combination offers a convenient all-oral regimen that combines the efficacy of a proteasome inhibitor and an immunomodulatory drug-based regimen for the treatment of NDMM with a well-tolerated and manageable toxicity profile, including a relatively low risk of PN, and the convenience of a reduced need for office visits. In patients not proceeding to SCT and in patients proceeding to maintenance therapy, the regimen can be continued long-term, with a manageable toxicity profile, and further allows for maintenance with single-agent ixazomib with no significant cumulative toxicities.

## Supplementary information

Supplementary Information

Supplementary Figure 1
