# Supplementary material for: Ixazomib, lenalidomide, and dexamethasone in patients with newly diagnosed multiple myeloma: long-term follow-up including ixazomib maintenance
Source: Leukemia. 2019 Jan 29;33(7):1736–46. doi: 10.1038/s41375-019-0384-1 (PMC6755968; doi:10.1038/s41375-019-0384-1)

**Supplementary Information**

**Supplementary Table S1:** Detailed eligibility criteria

**Supplementary Table S2:** Overall safety profile and summary of AEs in patients aged ≥65 years

**Supplementary Figure 1.** Mean EORTC QLQ-C30 global health status/QoL score in phase 2 patients who did not proceed to SCT (N=36)

**Supplementary Table S1.** Detailed eligibility criteria

| **Inclusion criteria** | **Exclusion criteria** |
| --- | --- |
| - Male or female patients aged ≥18 years - Symptomatic MM or asymptomatic myeloma with myeloma-related organ damage diagnosed according to standard criteria - Patients must have measurable disease defined by at least 1 of the following measurements:   - Serum M-protein ≥1 g/dL (≥ 10 g/L)   - Urine M-protein ≥200 mg/24 hours   - Serum free light chain assay: involved free light chain level ≥10 mg/dL (≥100 mg/L) provided the serum free light chain ratio is abnormal - Patients must meet the following clinical laboratory criteria as specified below within 3 days before the first dose of study treatment to be enrolled in the study:   - Absolute neutrophil count (ANC) ≥1,000/mm^3^ and platelet count ≥75,000/mm^3^ (platelet transfusions to help patients meet eligibility criteria not allowed within 3 days prior to study drug dosing)   - Total bilirubin <1.5 X upper limit of normal (ULN)   - Alanine aminotransferase (ALT) and aspartate aminotransferase (AST) <3 X ULN   - Calculated creatinine clearance ≥30 mL/min - ECOG performance status of 0 to 2 - Female patients who:   - are postmenopausal for at least 1 year before the screening visit   - are surgically sterile   - if they are of childbearing potential, agree to practice 2 effective methods of contraception, at the same time, from the time of signing the informed consent through 30 days after the last dose of study treatment, or agree to completely abstain from heterosexual intercourse   - must also adhere to the guidelines of the lenalidomide pregnancy prevention program - Male patients, even if surgically sterilised (i.e., status post-vasectomy), who:   - agree to practice effective barrier contraception during the entire study treatment period and through 4 months after the last dose of study treatment if their partner is of childbearing potential, even if they have had a successful vasectomy   - agree to completely abstain from heterosexual intercourse   - must also adhere to the guidelines of the lenalidomide pregnancy prevention program - Must be able to take concurrent aspirin 325 mg daily (or enoxaparin 40 mg subcutaneously daily [or its equivalent] if allergic to aspirin) as prophylactic anticoagulation - Voluntary written consent must be given before performance of any study related procedure not part of standard medical care, with the understanding that consent may be withdrawn by the patient at any time without prejudice to future medical care | - PN grade ≥2 on clinical examination during the screening period - Female patients who are lactating or pregnant - Major surgery within 14 days before the first dose of study drug - Infection requiring systemic antibiotic therapy or other serious infection within 14 days before the first dose of study drug - Diarrhea grade ≥1, based on the NCI CTCAE grading, in the absence of antidiarrheals - Prior systemic therapy for MM, including investigational proteasome inhibitors and iMIDs. Prior treatment with corticosteroids or localized radiation therapy does not disqualify the patient (maximum dose of corticosteroids should not exceed the equivalent of 160 mg of dexamethasone, a total of which can be given in a 2-week period) - Radiotherapy within 14 days before the first dose of study treatment - Systemic treatment with strong inhibitors of CYP1A2 (fluvoxamine, ciprofloxacin, enoxacin), strong inhibitors of CYP3A (clarithromycin, telithromycin, itraconazole, voriconazole, ketoconazole, nefazodone, posaconazole) or strong CYP3A inducers (rifampin, rifapentine, rifabutin, carbamazepine, phenytoin, phenobarbital), or use of Ginkgo biloba or St. John’s wort within 14 days before the first dose of study treatment - Central nervous system involvement - Evidence of current uncontrolled cardiovascular conditions, including uncontrolled hypertension, uncontrolled cardiac arrhythmias, symptomatic congestive heart failure, unstable angina, or myocardial infarction within the past 6 months - Prior or concurrent deep vein thrombosis or pulmonary embolism - Rate-corrected QT interval of electrocardiograph (QTc) > 470 milliseconds on a 12-lead electrocardiogram obtained during the screening period; if a machine reading is above this value, the electrocardiogram should be reviewed by a qualified reader and confirmed on a subsequent electrocardiogram - Known human immunodeficiency virus positive - Known hepatitis B surface antigen-positive status, or known or suspected active hepatitis C infection - Any serious medical or psychiatric illness that could, in the investigator’s opinion, potentially interfere with the completion of treatment according to this protocol - Known allergy to any of the study medications, their analogues, or excipients in the various formulations - Known gastrointestinal disease or gastrointestinal procedure that could interfere with the oral absorption or tolerance of study drug, including difficulty swallowing - Diagnosed or treated for another malignancy within 2 years before the first dose or previously diagnosed with another malignancy and have any evidence of residual disease. Patients with non-melanoma skin cancer or carcinoma in situ of any type are not excluded if they have undergone complete resection |

**Supplementary Table S2.** Overall safety profile and summary of AEs in patients aged ≥65 years

| **n (%)** | **Total N=34** | **Patients who received maintenance N=16** | **Patients who did not receive maintenance N=18** |
| --- | --- | --- | --- |
| Any AE | 34 (100) | 16 (100) | 18 (100) |
| Grade ≥3 AE | 27 (79) | 12 (75) | 15 (83) |
| Drug-related AE | 34 (100) | 16 (100) | 18 (100) |
| Drug-related grade ≥3 AE | 25 (74) | 11 (69) | 14 (78) |
| Serious AE | 14 (41) | 7 (44) | 7 (39) |
| Drug-related serious AE | 8 (24) | 4 (25) | 4 (22) |
| AE resulting in study drug dose reduction | 21 (62) | 12 (75) | 9 (50) |
| AE resulting in study drug discontinuation | 6 (18) | 0 (0) | 6 (33) |
| On-study deaths | 2 (6) | 0 (0) | 2 (11) |

**Supplementary Figure 1.** Mean EORTC QLQ-C30 global health status/QoL score in phase 2 patients who did not proceed to SCT (N=36)

BL, baseline; CI, confidence interval; EOT, end of treatment.


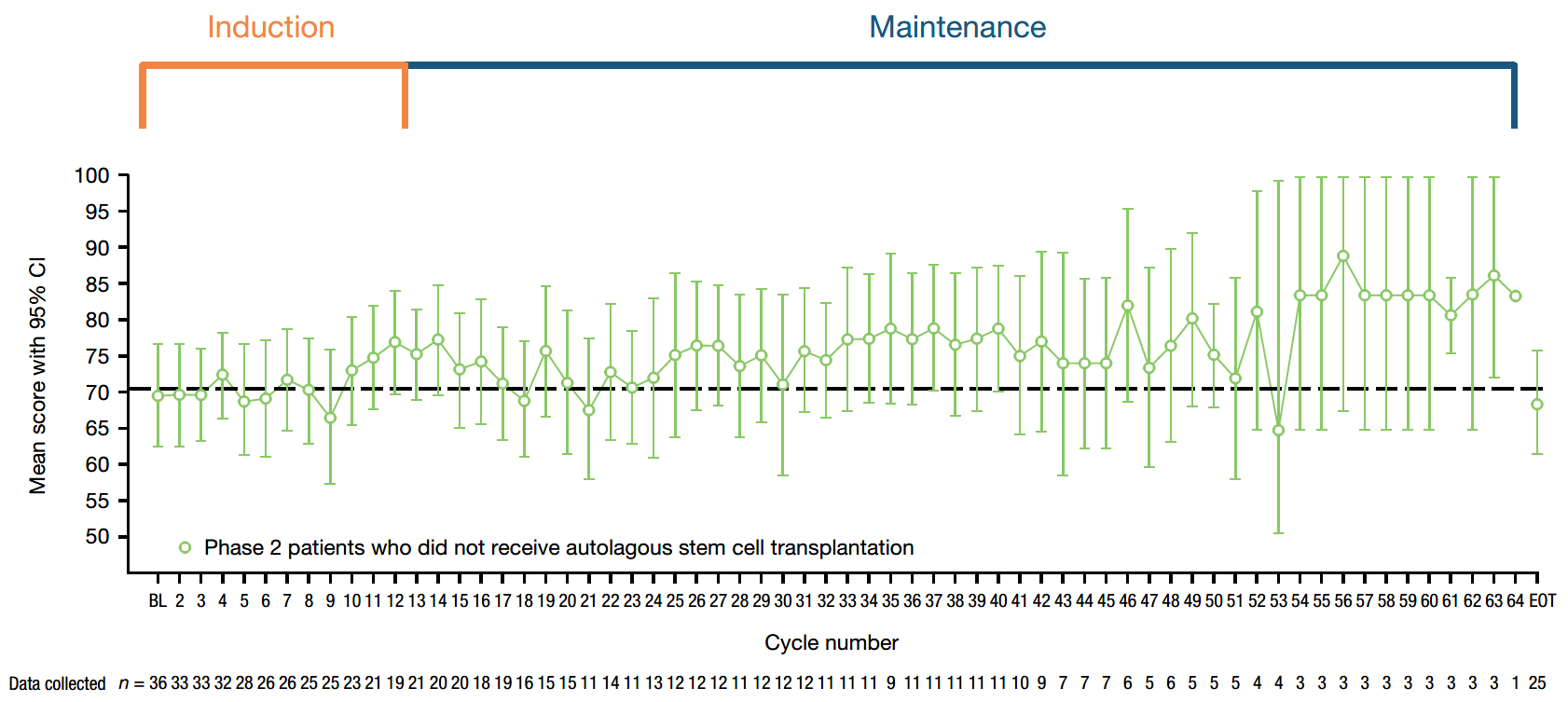

Supplement: Supplementary file 1 — Supplementary Information [file 41375_2019_384_MOESM1_ESM.docx]
