# Supplementary figures and images for: Ixazomib, lenalidomide, and dexamethasone in patients with newly diagnosed multiple myeloma: long-term follow-up including ixazomib maintenance
Source: Leukemia. 2019 Jan 29;33(7):1736–46. doi: 10.1038/s41375-019-0384-1 (PMC6755968; doi:10.1038/s41375-019-0384-1)

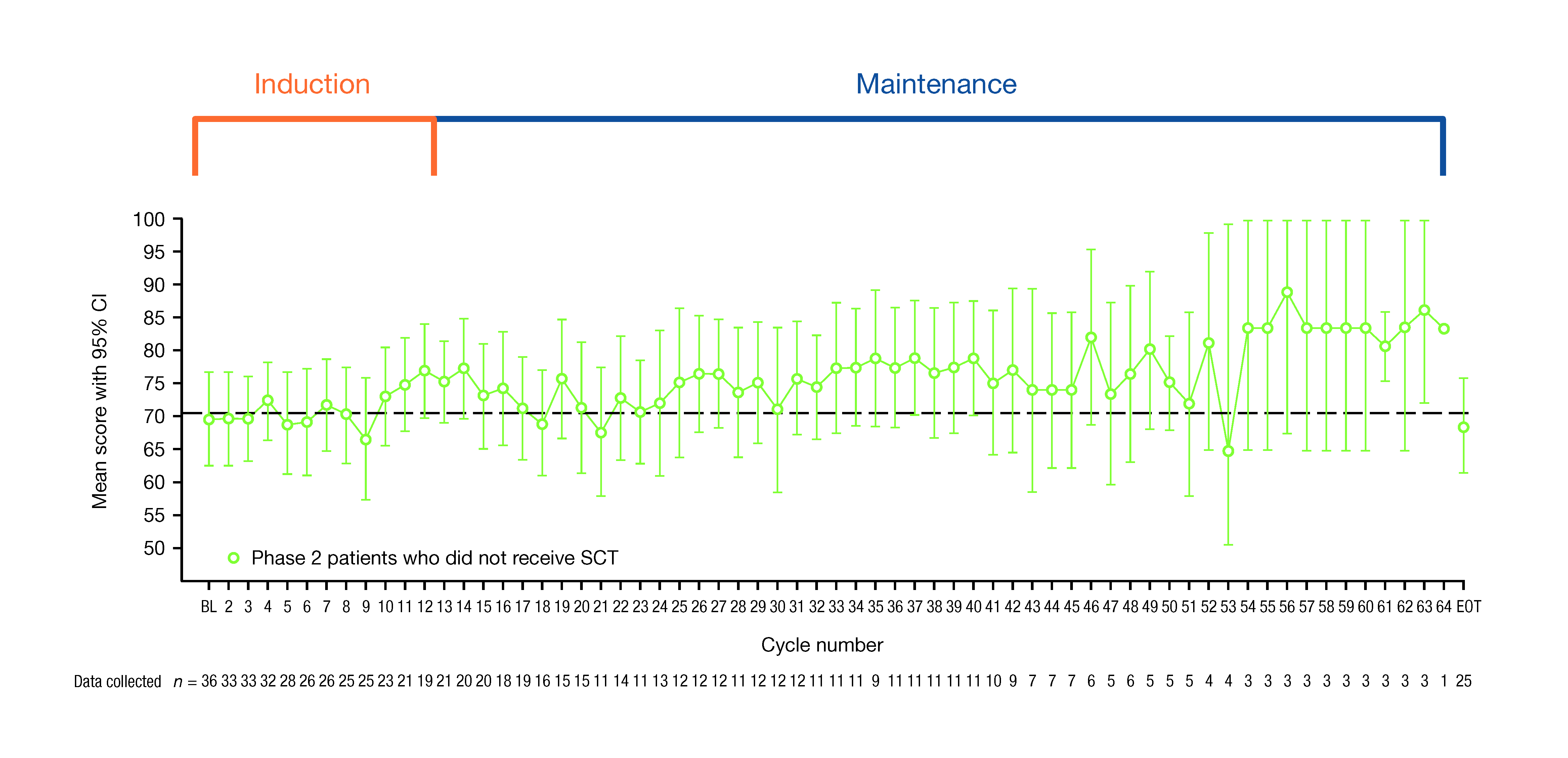

Supplement: Supplementary file 2 — Supplementary Figure 1 [file 41375_2019_384_MOESM2_ESM.tif]
